# Measuring empathy for human and robot hand pain using electroencephalography

**DOI:** 10.1038/srep15924

**Published:** 2015-11-03

**Authors:** Yutaka Suzuki, Lisa Galli, Ayaka Ikeda, Shoji Itakura, Michiteru Kitazaki

**Affiliations:** 1 Department of Computer Science and Engineering, Toyohashi University of Technology, Toyohashi, Japan; 2 Freie Universitaet Berlin, Berlin, Germany; 3 Department of Psychology, Graduate School of Letters, Kyoto University, Kyoto, Japan

## Abstract

This study provides the first physiological evidence of humans’ ability to empathize with robot pain and highlights the difference in empathy for humans and robots. We performed electroencephalography in 15 healthy adults who observed either human- or robot-hand pictures in painful or non-painful situations such as a finger cut by a knife. We found that the descending phase of the P3 component was larger for the painful stimuli than the non-painful stimuli, regardless of whether the hand belonged to a human or robot. In contrast, the ascending phase of the P3 component at the frontal-central electrodes was increased by painful human stimuli but not painful robot stimuli, though the interaction of ANOVA was not significant, but marginal. These results suggest that we empathize with humanoid robots in late top-down processing similarly to human others. However, the beginning of the top-down process of empathy is weaker for robots than for humans.

Empathy is one of the most important abilities for appropriate social communication. It allows us to understand others’ emotion and feelings even though we have not experienced the same situations^1^,^2^. Decety and Lamm (2006)^3^ proposed a model of empathy containing two levels of processing: bottom-up emotion sharing and top-down executive control to regulate empathy. Decety (2010)^2^ refined the model and proposed three components for empathy: affective arousal, emotion understating, and emotion regulation.

Numerous studies on emotion for pain have been reported, and painful situations induce strong empathic responses^4^. A functional magnetic resonance imaging study (fMRI) reported that electric pain stimuli activated the anterior insula (AI), anterior cingulate cortex (ACC), brain stem, and cerebellum both when the participants’ hands were stimulated and when they observed electric pain stimulation applied to their partners’ hands^5^. Similar brain activities occur when participants observe a facial expression of pain or a body part that receives painful stimuli^6^,^7^,^8^,^9^. Recent studies have suggested that brain regions including the bilateral AI, dorsal anterior cingulate cortex (dACC), anterior midcingulate cortex (aMCC), somatosensory cortex, and inferior frontal gyrus (IFG) play key roles in empathy for others in pain^4^,^5^,^6^,^8^,^10^,^11^,^12^,^13^,^14^,^15^.

Electroencephalography (EEG) studies have revealed temporal aspects of neural processing of empathy for pain^16^,^17^,^18^. Observation of painful photographic pictures induces a positive shift of brain potentials at 110 ms and later after stimulus presentation (early component), and larger P3 amplitudes than non-painful pictures (late component). The early component decreases when cartoon (non-photorealistic) pictures are used but is not affected by participants’ attention to the stimulus. In contrast, the late component decreases when participants do not pay attention to pain, but is not affected by picture reality^16^. These findings support the two-level (early bottom-up and late top-down processing) theory of Decety and Lamm (2006)^3^. The differential P3 effect for painful and non-painful pictures is larger for female than for male participants, and a significant negative correlation between the early negative component and the subjective painful stimulus rating is only observed for females^19^. Event-related potential (ERP) components induced by painful pictures are influenced by various factors, such as attentional demands^16^, perspective taking^18^, medical training^17^, racial bias^20^,^21^, duration of stimulus exposure^22^, social context^23^, preceding presentation of another’s face picture^24^, and emotional pictures^25^. Thus, ERP is a good tool to investigate various factors of empathy, particularly in temporal aspects.

Robots are becoming more popular and familiar in our everyday lives. Thus, we need to understand how humans socially interact with robots^26^. It is known that humans naturally engage in social communications with computers and virtual characters^27^,^28^,^29^. The “media equation” phenomenon may also be applicable to robots^30^,^31^. If this is the case, we can empathize with both robots and humans. There have been few studies on empathy for pain experienced by non-human others, such as robots and animals. One study^32^ reported significantly higher right limbic system activities in subjects observing violent human-human interactions compared to violent human-robot interactions. Their results suggest that we show similar neural activities for empathy directed toward humans and robots, but there are some differences in empathic neural response between empathy for humans and robots. Behavioral studies report that infants have abilities for empathy with non-human agents, such as geometrical objects^33^,^34^,^35^. Thus, we focused on the empathy with robots and predicted that human observers could empathize with robots, as well as human others, but with some differences.

The purpose of our study was to investigate neural responses to empathy for pain of a robot hand in comparison with a human hand by measuring ERPs during a well-established empathy-for-pain experimental paradigm^6^,^8^,^9^,^16^,^18^,^19^,^22^,^23^,^24^,^25^,^36^,^37^,^38^. To our knowledge, there has been no study on empathy for robot pain using EEG. We hypothesized that similar ERPs would be found for both human and robot painful stimuli if we indeed empathize with robots. Moreover, we aimed to investigate different processes underlying responses to human and robot with regard to early bottom-up and late top-down processing.

## Results

**Figure 1:**
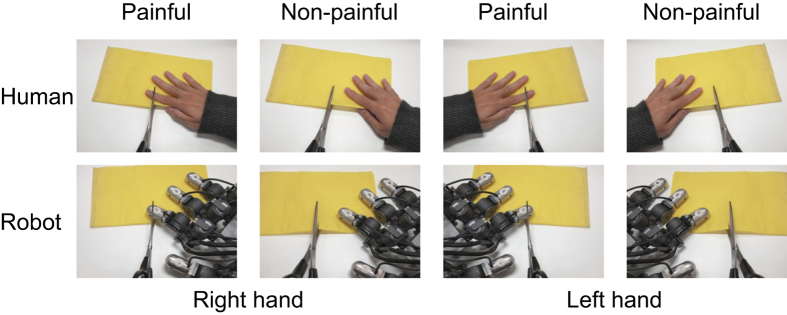
Example of stimuli used in the experiment. Luminance of painful and non-painful pictures was equalized.

We measured event-related potentials of human participants who were observing pictures of painful or non-painful situations with human hands or robot hands (Fig. 1).

### ERP data

**Figure 2:**
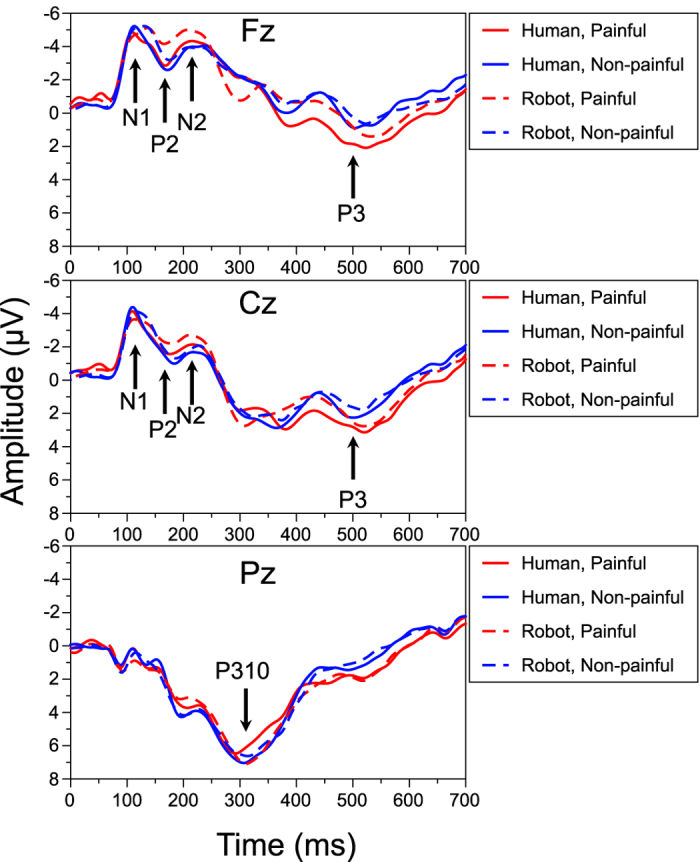
Grand averages of EEG at Fz, Cz, and Pz. Abscissa is time after stimulus onset. Ordinate is amplitude of brain potentials. ERP components (N1, P2, N2, P310, P3) are shown in each graph.

We defined the N1, P2, N2, P310, and P3 components based on the grand-averaged values at each channel by visual inspection (Fig. 2). We analyzed P3 with division into the former half (ascending phase of P3) and the latter half (descending phase of P3). The mean amplitude was calculated from each time window at appropriate channels as follows: N1: 90–120 ms at Fz and Cz, P2: 140–190 ms at Fz and Cz, N2: 190–250 ms at Fz and Cz, P310: 300–350 ms at Pz, ascending phase of P3: 350–500 ms at Fz and Cz, descending phase of P3: 500–650 ms at Fz and Cz. The nomenclature of ERP components at 350-650 ms as P3 is based on the previous studies^16^,^18^ on empathy for pain. We used the mean amplitudes for ERPs rather than peak amplitudes because previous studies^16^,^17^,^18^,^19^ using similar stimuli and a similar paradigm employed the mean amplitude. Averaged ERPs were calculated from 101–112 trials for each condition after artifact rejection (98.25% of all trials on average). We conducted a two-way repeated-measure analyses of variance (ANOVAs) with the factors of Model (human vs. robot), Pain (painful vs. non-painful), and Electrode (Fz, Cz) for N1, P2, N2, ascending phase of P3, and descending phase of P3, and a one-way repeated-measure ANOVA with the factors of Model (human vs. robot) and Pain (painful vs. non-painful) for P310 as within-subject independent variables. We applied the FDR (false discovery rate, q = 0.05) of Benjamini and Hockberg^39^ for post-hoc multiple comparisons of simple main effects.

### N1 components (90–120 ms)

There were no significant interactions (Model × Pain: F[1,14] = 0.530, p = 0.479, η_p_^2^ = 0.037; Model × Electrodes: F[1,14] = 0.957, p = 0.088, η_p_^2^ = 0.194; Pain × Electrodes: F[1,14] < 0.001, p = 0.996, η_p_^2^ < 0.001; Model × Pain × Electrodes: F[1,14] = 0.040, p = 0.846, η_p_^2^ = 0.028). The main effect of Electrodes was significant (F[1,14] = 28.810, p < 0.001, η_p_^2^ = 0.673). The other main effects were not significant (Model: F[1,14] = 1.863, p = 0.194, η_p_^2^ = 0.117; Pain: F[1,14] = 0.699, p = 0.417, η_p_^2^ = 0.048). These results revealed that the N1 component was not influenced by Pain or Model effects.

### P2 components (140–190 ms)

The Model × Electrodes interaction was marginally significant (F[1,14] = 3.890, p = 0.069, η_p_^2^ = 0.218). The simple main effect of Model was only significant in Fz (F[1,14] = 11.404, p = 0.005, η_p_^2^ = 0.449) and not significant in Cz (F[1,14] = 2.914, p = 0.110, η_p_^2^ = 0.172). These results indicated that human stimuli induced a larger positive shift of P2 (140–190 ms) than robot stimuli at Fz.

### N2 components (190–250 ms)

**Figure 3:**
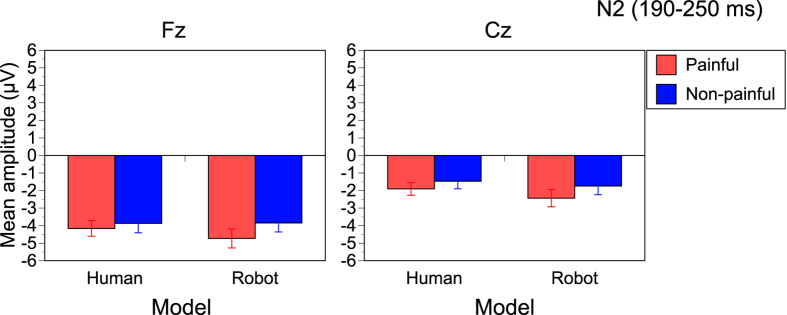
Average amplitudes of N2 (190–250 ms). N2 was larger with painful stimuli than non-painful stimuli. The human stimuli induced a positive shift of the N2 amplitude than robot stimuli.

We found a significant main effect of Pain (F[1,14] = 8.039, p = 0.013, η_p_^2^ = 0.365), indicating that painful stimuli elicited larger N2 than non-painful stimuli, irrespective of Model or Electrodes (Fig. 3). This effect was inconsistent with a positive shift of N2 for human pain in a previous study^16^. There were no significant interactions. The main effect of Electrode was significant (F[1,14] = 38.732, p < 0.001, η_p_^2^ = 0.735), and the main effect of Model was marginally significant (F[1,14] = 4.012, p = 0.065, η_p_^2^ = 0.223). Human stimuli induced a positive shift of N2 (190–250 ms) compared to robot stimuli, similarly to P2.

### P310 components (300–350 ms)

**Figure 4:**
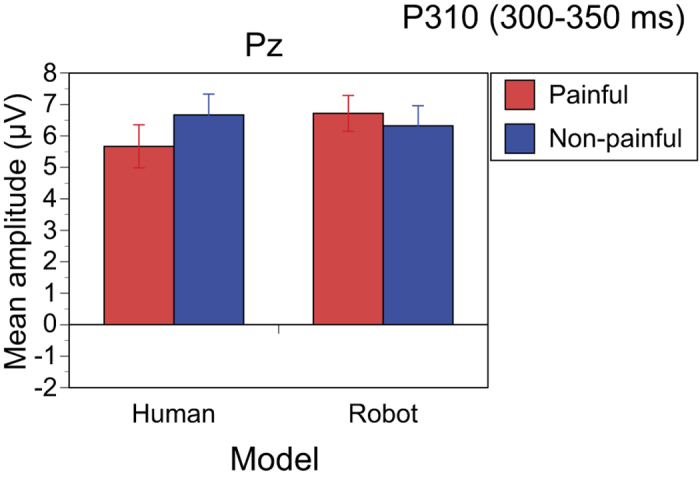
Average amplitudes of P310 (300–350 ms). P310 positively shifted with robot painful stimuli relative to human painful stimuli. Amplitude of P310 was less with human painful stimuli than human non-painful stimuli.

The Model × Pain interaction was significant (F[1,14] = 22.243, p < 0.001, η_p_^2^ = 0.614). The simple main effect of Model was only significant in the Painful condition (F[1,14] = 8.120, p = 0.013, η_p_^2^ = 0.367), indicating that robot painful stimuli elicited larger P310 amplitudes than human painful stimuli (Fig. 4). The simple main effect of Pain was only significant in the Human condition (F[1,14] = 8.071, p = 0.013, η_p_^2^ = 0.366), indicating that human non-painful stimuli elicited larger P310 amplitudes than human painful stimuli.

### Ascending phase of P3 (350–500 ms)

**Figure 5:**
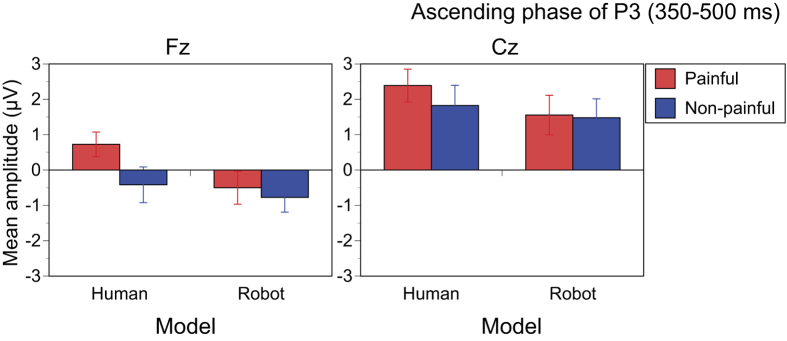
Average amplitudes of the ascending phase of P3 (350–500 ms). The ascending phase of P3 was larger in painful than in non-painful stimuli of human, but not of robots.

Main effects of Pain (F(1,14) = 7.033, p = 0.019, η_p_^2^ = 0.334), Model (F(1,14) = 19.402, p < 0.001, η_p_^2^ = 0.581), and Electrode (F(1,14) = 41.487, p < 0.001, η_p_^2^ = 0.748) were significant. The Pain × Electrodes interaction was significant (F[1,14] = 8.782, p = 0.010, η_p_^2^ = 0.386). The simple main effect of Pain was only significant at Fz (F[1,14] = 11.721, p = 0.004, η_p_^2^ = 0.456), indicating that the ascending phase of late P3 recorded at Fz was increased by painful stimuli more than by non-painful stimuli (Fig. 5). The Pain × Model interaction approached significance (F[1,14] = 3.580, p = 0.079, η_p_^2^ = 0.204). The simple main effect of Pain was significant for human hands (F[1,14] = 7.168, p = 0.018, η_p_^2^ = 0.339) but not for robot hands (F[1,14] = 0.819, p = 0.381, η_p_^2^ = 0.055), indicating that the ascending phase of late P3 was shifted positively by painful stimuli only for human stimuli.

### Descending phase of P3 (500–650 ms)

**Figure 6:**
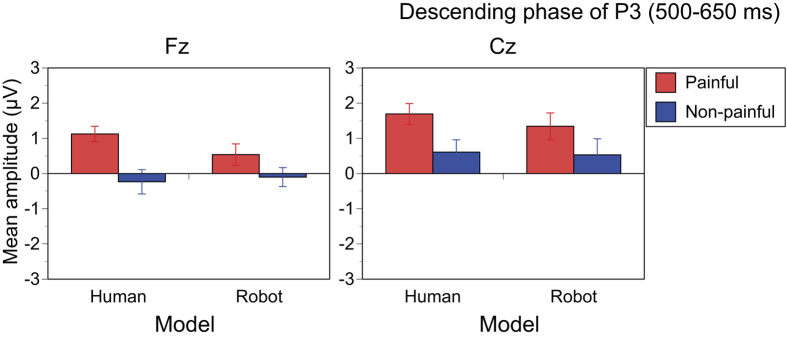
Average amplitudes of the descending phase of P3 (500–650 ms). The descending phase of P3 was larger in painful than in non-painful stimuli of both human and robots.

Main effects of Pain (F[1,14] = 29.472, p < 0.001, η_p_^2^ = 0.678) and Electrode (F[1,14] = 11.250, p = 0.005, η_p_^2^ = 0.446) were significant, but that of Model was not significant (F[1,14] = 1.192, p = 0.293, η_p_^2^ = 0.079). The Pain × Model interaction was not significant (F[1,14] = 2.030, p = 0.176, η_p_^2^ = 0.127), and the other interactions were also not significant. Thus, painful stimuli elicited a larger descending phase of P3 than non-painful stimuli, irrespective of whether the hand belonged to a human or robot (Fig. 6).

### Correlation between ERP amplitudes and personal empathic ability

We calculated the mean differential ERP amplitudes between painful and non-painful conditions and performed a correlation analysis between those values and subjects’ IRI (Interpersonal Reactivity Index)^40^ scores and EQSQ (Empathizing Quotient Systemizing Quotient)^41^ scores. We did not find any significant correlation with either Bonferroni correction or FDR.

## Discussion

We investigated the effect of the visual appearance of the agent (human and robot) on ERPs induced by empathy for pain. We found common neural responses for empathy directed toward humans and robots in the descending phase of P3 (500–650 ms), and a human-specific response in the ascending phase of P3 (350–500 ms). The descending phase of P3 components was larger for the painful stimuli than for the non-painful stimuli, regardless of whether the hand belonged to a human or robot. In contrast, the ascending phase of the P3 component at the frontal-central electrodes was increased by painful human stimuli but not painful robot stimuli, although the statistical significance was marginal.

Similar to previous studies, our results showed that the P3 amplitude was greater when participants observed human others in painful situations relative to non-painful situations^16^,^17^,^18^,^19^,^21^,^22^,^23^,^24^,^25^,^38^. A important new finding was that the pain effect (differential amplitude between painful and non-painful conditions) shown in the ascending phase of P3 (350–500 ms) was only significant for human stimuli, but in the descending phase of P3 (500–650 ms), it was significant for both human and robot stimuli. These findings suggest that visual cues for humanity (human or robot) partially modulate the top-down controlled processes of empathy for pain, but human observers fundamentally show empathic neural responses to robots, similarly to human others.

Li and Han (2010)^18^ reported that the ascending phase of the P3 (370–420 ms) response to others in painful situations decreased when participants were told to take the other’s perspective while performing a pain judgment task, but the descending phase of P3 response was not influenced by perspective taking^18^. These results were similar to our findings; ERP modulation by visual cues of humanity was found in the ascending phase of P3 (350–500 ms) but not in the descending phase of P3 (500–650 ms). Therefore, we speculate that the difference in empathy between human and robot others was related to perspective taking, which was more difficult for robot others. Because there was no difference in the pain effect between humans and robots at the descending phase of P3 (500–650 ms), it is suggested that emotional regulation, which is reflected in LPP after 600 ms^22^,^42^,^43^, functions similarly with respect to robots and humans. Thus, humans can attribute humanity to robots and feel their pain. Because the basic shape of the robot hand in the present study was the same as that of the human hand, the human participants may have been able to empathize with the robot hand. It is thus necessary to test whether a robot hand in very different shape (e.g., a robot hand without fingers) can elicit similar empathic responses in a future study.

We did not find any effect of pain conditions on N1 at 90–120 ms or P2 at 140–190 ms. It is controversial whether the N1 (N110) amplitude is influenced by empathy for pain. Recent ERP studies suggested that an early empathic response induced a positive shift of the N1 (N110) component^16^,^17^,^19^,^24^. However, several studies suggested that the N1 (N110) amplitude was not influenced by empathy for others in pain^21^,^23^,^37^, which is consistent with the present study.

We found a significant pain effect on N2 at 190–250 ms, which was larger for painful stimuli than for non-painful stimuli, irrespective of whether the hand belonged to a human or robot. This finding conflicts with the previous studies, which show a negative shift of N2 in painful situations.

We found a main effect of model (human and robot) at both P2 and N2, but we did not observe an interaction between model and pain. Thus, differences in hand appearance or visual cues for humanity would modulate very early brain potentials. These results suggest that visual humanity coding occurs in early visual processing.

The P310 (300–350 ms) amplitudes were greater for robot painful stimuli than human painful stimuli, and greater for human non-painful stimuli than human painful stimuli. In other words, P310 was weaker for the human painful stimuli than for the robot painful and the human non-painful stimuli. Fan and Han (2008)^16^ reported that the ERP amplitude at 220–300 ms around Pz was larger for non-painful human stimuli than for painful human stimuli. Their result is similar to our findings, although their time window (220–300 ms) was earlier than ours (300–350 ms). The ERP component around 300 ms reflects the process of detecting an infrequent target, usually in the oddball paradigm. It is modulated not only by stimulus frequency, but also by unnaturalness of visual stimuli^44^. The difference between the human painful hand and the robot painful hand may be explained by the unnaturalness of robot hands cut by knives.

It may be argued that the weak response in the ascending phase of P3 with the robot hand may have been caused by differences in stimulus contrast and size between the human and robot hands in pictures. It is reported that pain perception is modulated by the visual size of the body^45^. We carefully controlled stimulus luminance between the painful and non-painful stimuli for both human and robot hands, but did not control the contrast or size between human and robot hands. Then, we conducted a perceptual experiment to measure reaction times to judge pain or lack of pain in pictures with the human- and robot-hand stimuli identical to those of the main experiment. We found a main effect of the human and robot hands in reaction times; the accelerated judgments were faster for the human hands than for the robot hands. Therefore the pain discrimination was easier for the human-hand stimuli than for the robot-hand stimuli, possibly because the robot stimuli had low color contrast and the robot hand was larger than the human hand, although the knives were identical. However, it is not reasonable to consider that the different response of the ERP in the ascending phase of P3 between the human and the robot hands was caused by differing visibility of the stimuli. Because the reality of the stimuli induces a pain effect in N2 (the early component) but not in the late components^16^, the visibility also should affect the early processing and not late processing such as P3. The effect of the visual appearance of the human or robot hand on the pain condition was limited to the ascending phase of P3 and not found in N2 or the descending phase of P3 in the present study. This selective effect cannot be explained solely by stimulus visibility.

Our study showed that humans empathize with both humans and humanoid robots during late top-down processing, as confirmed by identical pain effects in the descending phase of P3. However, there was a difference in the beginning of the top-down-controlled processing of empathy. The pain effect of the ascending phase of P3 was only significant when empathy was directed toward humans, possibly because of the difficulty in taking the perspective of robots. However, the interaction of the human-robot appearance condition and the painful condition by ANOVA was not significant, but just marginal. Thus, the conclusion regarding the ascending phase of P3 should be carefully considered on its limitation.

## Methods

### Participants

Fifteen undergraduate and graduate students (3 females and 12 males, mean 21.7 years old ± 1.4 standard deviation [SD], 1 left-handed and 14 right-handed) participated in the experiment. All participants provided written informed consent and had normal or corrected-to-normal vision. The methods of the experiment and all experimental protocols were approved by the Committee for Human-Subject Studies at Toyohashi University of Technology, and the experiment was strictly conducted in accordance with the approved guidelines of the committee.

### Stimuli and apparatus

Participants were shown 56 color photographs of a human or robot hand in painful or non-painful situations. The images were from a first-person perspective. The painful pictures illustrated accidents that may happen in everyday life, such as a finger cut by a knife, and the corresponding non-painful pictures were made by moving knife-like objects a small distance from the hand (Fig. 1). Seven different situations were used. Left-hand conditions were mirror-reversed pictures of right-hand stimuli. The luminances of painful and non-painful pictures were controlled to be identical (average 14.030 cd/m^2^). Stimulus presentation was controlled by a computer (Dell Vostro 420, Intel Core2Duo E8500 with 3.16 GHz, 4.0 GB RAM, ATI Radeon HD3400, running Microsoft Windows 7) and displayed at the center of a gray (9.018 cd/m^2^) background of a CRT color monitor (TOTOKU CV921X, 30 × 40 cm, 1024 × 768 pixels, 60-Hz refresh). Each stimulus was 20 × 15 cm, 512 × 384 pixels (11.42 × 8.58 deg at a viewing distance of 100 cm).

### Procedure

The ERP recording experiment consisted of eight sessions. Each session included 56 recording trials (56 different pictures: combinations of 7 situations, 2 model conditions (human or robot), 2 pain conditions (painful or non-painful), and left and right hand pictures) and 10 filler trials with pain judgment tasks in a random order. Filler trials were added to sustain participants’ attention to pain components in the stimulus and were removed before the ERP analysis. At the beginning of each trial, the stimulus was displayed at the center of the screen (500 ms) following a fixation cross presentation (1000 ms). If it was an ERP recording trial, the next trial continued after a blank screen (randomized at 900–1600 ms). If the trial was the filler, a question mark was presented after stimulus presentation, and participants judged whether the picture was painful or non-painful by pushing one of two buttons held in each hand. The next trial continued after a blank screen was presented (randomized at 1400–2100 ms). Correspondence between buttons and options was counter-balanced among the participants.

### Measurements of subjective ratings

After the ERP recording experiment, subjects re-observed all the stimuli without time restrictions. They were asked to provide two ratings for each stimulus: the intensity of pain supposedly felt by the model in the stimuli, and the unpleasantness they felt when they observed the picture. They responded using a mouse to select a value on the Visual Analog Scale on the monitor (approximated as 0–100). The subjects felt greater pain intensity and self-unpleasantness in the painful than in the non-painful conditions, and the difference between painful and non-painful conditions was larger with human-hand stimuli than with robot stimuli. Detailed results and statistical analysis are described in the Supplementary Materials.

### EEG recording

EEG signals were recorded by a biological amplifier (TEAC Polymate AP1132, 32 channel, 16 bit, up to 2000 Hz sampling) using 32 scalp electrodes (Neuroscan, Quik-Cap) according to the extended 10-20 system with two earlobe electrodes. The electrode at the left earlobe was used as reference. The electrode impedance was kept below 10  Ω. The EEG was amplified (band pass 0.5–30 Hz) and digitized at a sampling rate of 1000 Hz. The ERPs in each condition were averaged separately offline with an epoch beginning at stimulus onset and continuing for 700 ms. Trials containing eye blinks, eye movements, or muscle potentials exceeding ±50 μV were excluded from the average for each electrode.

### ERP analysis

EEG at each electrode was re-referenced to the algebraically computed average of the left and right earlobes. The mean amplitude of the 200 ms pre-stimulus interval was used as the baseline for each ERP measurement. The rejection rates of each electrode were as follows: (Fz: 2.798 ± 5.711, Cz: 0.655 ± 1.170, Pz: 0.580 ± 1.282 [%]).

### Measures of participants’ empathy characteristics

Before the ERP recording experiment, participants filled out two questionnaires including the interpersonal reactivity index (IRI)^40^ and the Empathizing Quotient Systemizing Quotient (EQSQ)^41^ to measure personal empathy ability. The Japanese versions of both questionnaires were used^46^,^47^. IRI scores of the present participants were similar (difference within 1.0) to those of control participants of Decety *et al.* (2010)^17^ except for the empathic concern subscale (5.93 lower than noted by Decety *et al.*, 2010).

## Additional Information

**How to cite this article**: Suzuki, Y. *et al.* Measuring empathy for human and robot hand pain using electroencephalography. *Sci. Rep.* **5**, 15924; doi: 10.1038/srep15924 (2015).

## Supplementary Material

Supplementary Information
