# Supplementary material for: Measuring empathy for human and robot hand pain using electroencephalography
Source: Sci Rep. 2015 Nov 3;5:15924. doi: 10.1038/srep15924 (PMC4630641; doi:10.1038/srep15924)
Supplement: Supplementary Information [file srep15924-s1.pdf]

Measuring empathy for human and robot hand pain using electroencephalography

Yutaka Suzuki[1], Lisa Galli[2], Ayaka Ikeda[3], Shoji Itakura[3], Michiteru Kitazaki[1*]

(1) Department of Computer Science and Engineering, Toyohashi University of Technology, Toyohashi, Japan

(2) Freie Universitaet Berlin, Berlin, Germany

(3) Department of Psychology, Graduate School of Letters, Kyoto University, Kyoto, Japan

*Correspondence:

Michiteru Kitazaki

Email: mich@cs.tut.ac.jp

Department of Computer Science and Engineering,

Toyohashi University of Technology

1-1 Hibarigaoka, Tempaku-cho, Toyohashi, Aichi 441-8580, Japan

Phone: +81-532-44-6889 Fax: +81-532-44-6889

Supplementary materials

Results of subjective ratings: The subjects felt greater pain intensity and self-unpleasantness in the painful than in the non-painful condition for both human and robot hands, and the difference between painful and non-painful conditions was larger with human-hand stimuli than with robot stimuli (Figure S1). We performed three-way repeated measures ANOVAs with the factors of Hand (Right vs. Left), Model (Human vs. Robot), and Pain (Painful vs. Non-painful) using the pain intensity rating and the self-unpleasantness rating.

The ANOVA on pain intensity rating showed main effects of Model ($F[1,14] = 19.772$, $p < 0.001$, $\eta_p^2 = 0.586$) and Pain ($F[1,14] = 157.60$, $p < 0.001$, $\eta_p^2 = 0.918$), and a significant Model × Pain interaction ($F[1,14] = 17.454$, $p < 0.001$, $\eta_p^2 = 0.555$). Post-hoc analysis showed significant simple main effects of Pain in both human and robot conditions (human: $F[1,14] = 358.86$, $p < 0.001$, $\eta_p^2 = 0.963$; robot: $F[1,14] = 39.305$, $p < 0.001$, $\eta_p^2 = 0.737$), indicating that pain intensity was greater in the painful than in the non-painful condition. The simple effect of Model was significant in the

painful condition but not in the non-painful condition (Painful: F[1,14] = 18.780, $p < 0.001$, $\eta_p^2 = 0.573$; Non-painful: F[1,14] = 0.7807, $p = 0.392$, $\eta_p^2 = 0.005$), indicating that human stimuli induced higher pain feelings in observers than robot stimuli.

For self-unpleasantness ratings, the main effects of Model (F[1,14] = 13.461, $p = 0.003$, $\eta_p^2 = 0.490$) and Pain (F[1,14] = 123.28, $p < 0.001$, $\eta_p^2 = 0.898$) and the interaction of Model × Pain (F[1,14] = 7.723, $p = 0.015$, $\eta_p^2 = 0.356$) were significant. The post-hoc analysis showed simple main effects of Pain in Human (F[1,14] = 170.05, $p < 0.001$, $\eta_p^2 = 0.924$) and Robot conditions (F[1,14] = 43.566, $p < 0.001$, $\eta_p^2 = 0.757$). These results indicated that painful stimuli elicited higher unpleasantness in observers than non-painful stimuli. The simple main effects of Model were significant in both the Painful and Non-painful conditions (Painful: F[1,14] = 11.353, $p = 0.005$, $\eta_p^2 = 0.448$; Non-painful: F[1,14] = 5.093, $p = 0.041$, $\eta_p^2 = 0.267$). These results indicated that human stimuli elicited higher unpleasantness in observers than robot stimuli, irrespective of Pain or Non-painful conditions, but the effect size was larger in the painful condition. We found a significant main effect of Hand (F[1,14] = 5.175, $p = 0.039$, $\eta_p^2 = 0.270$), indicating that right-hand stimuli induced higher unpleasantness in

observers than left-hand stimuli.

Figure captions

Figure S1: Subjective ratings of pain-intensity (top) and self-unpleasantness (bottom) of stimuli.

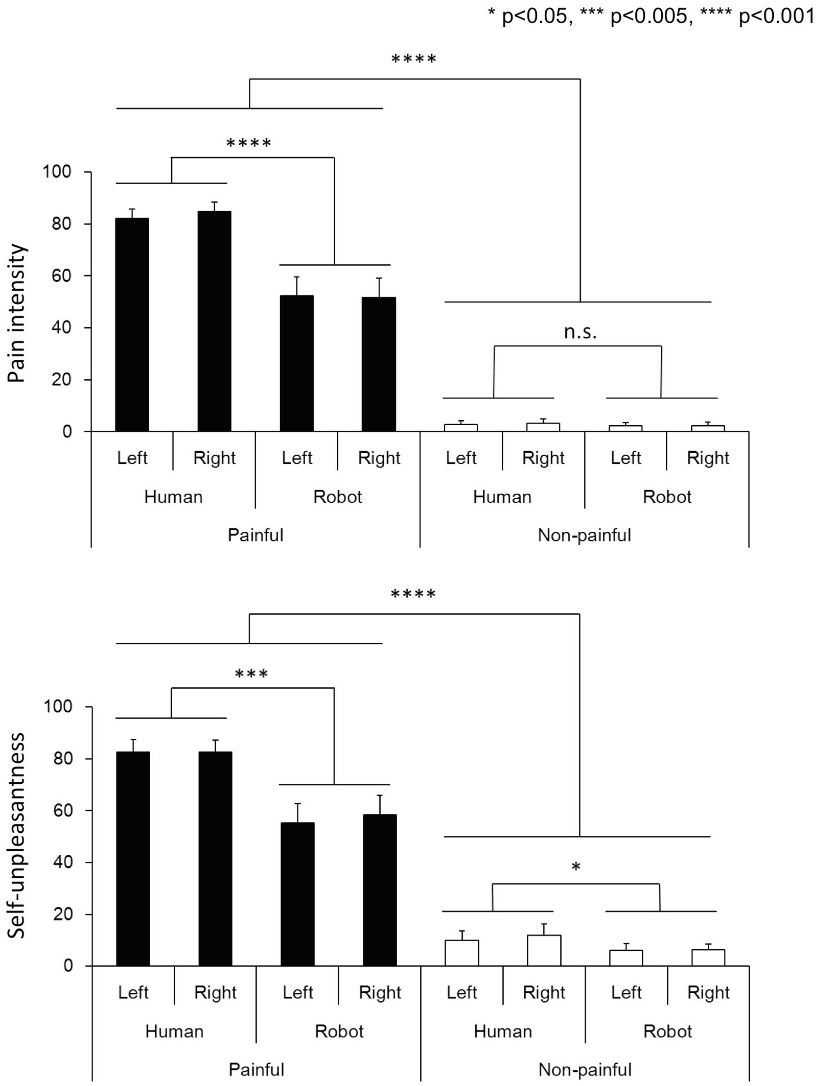

Figure S1
